# Quality of governance, public spending on health and health status in Sub Saharan Africa: a panel data regression analysis

**DOI:** 10.1186/s12889-015-2287-z

**Published:** 2015-09-21

**Authors:** Innocent Makuta, Bernadette O’Hare

**Affiliations:** University of Malawi, Chancellor College, Zomba, Malawi; University of Malawi, The Polytechnic, Blantyre, Malawi; School of Medicine, University of St Andrews, Fife, Scotland; School of Public Health and Family Medicine, College of Medicine, University of Malawi, Blantyre, Malawi

**Keywords:** Public health spending, Sub Saharan Africa, Under -five mortality, Life expectancy, Governance, Corruption

## Abstract

**Background:**

The population in Sub Saharan Africa (SSA) suffers poor health as manifested in high mortality rates and low life expectancy. Economic growth has consistently been shown to be a major determinant of health outcomes. However, even with good economic growth rates, it is not possible to achieve desired improvements in health outcomes. Public spending on health (PSH) has long been viewed as a potential complement to economic growth in improving health. However, the relationship between PSH and health outcomes is inconclusive and this inconclusiveness may, in part, be explained by governance-related factors which mediate the impact of the former on the latter. Little empirical work has been done in this regard on SSA. This paper investigates whether or not the quality of governance (QoG) has a modifying effect on the impact of public health spending on health outcomes, measured by under-five mortality (U5M) and life expectancy at birth (LE), in SSA.

**Methods:**

Using two staged least squares regression technique on panel data from 43 countries in SSA over the period 1996–2011, we estimated the effect of public spending on health and quality of governance U5M and LE, controlling for GDP per capita and other socio-economic factors. We also interacted PSH and QoG to find out if the latter has a modifying effect on the former’s impact on U5M and LE.

**Results:**

Public spending on health has a statistically significant impact in improving health outcomes. Its direct elasticity with respect to under-five mortality is between −0.09 and −0.11 while its semi-elasticity with respect to life expectancy is between 0.35 and 0.60. Allowing for indirect effect of PSH spending via interaction with quality of governance, we find that an improvement in QoG enhances the overall impact of PSH. In countries with higher quality of governance, the overall elasticity of PSH with respect to under-five mortality is between −0.17 and −0.19 while in countries with lower quality of governance, it is about −0.09. The corresponding semi elasticities with respect to life expectancy are about 6 in countries with higher QoG and about 3 in countries with lower QoG.

**Discussion:**

Public spending on health improves health outcomes. Its impact is mediated by quality of governance, having the higher impact on health outcomes in countries with higher quality of governance and lower impact in countries with lower quality of governance. This may be due to increased efficiency in the use of available resources and better allocation of the same as QoG improves.

**Conclusion:**

Improving QoG would improve health outcomes in SSA. The same increase in PSH is twice as effective in reducing U5M and increasing LE in countries with good QoG when compared with countries with poor QoG.

## Background

The population in Sub Saharan Africa (SSA) suffers poor health as manifested in high mortality rates and low life expectance at birth, indicators widely used as aggregate measures of a population’s health status. Life expectancy at birth (LE) in SSA is at 57 years, the lowest across all regions of the world. Under-five mortality rate (U5M) and maternal mortality ratio (MMR) in SSA are the highest in the world at 98 per 1000 live births and 510 per 100,000 respectively [1]. Both of these are twice as high as the (arithmetic) mean for the developing countries which stand at 53 per 1000 and 230 per 100,000 respectively. Hence, the region bears the bulk of global child and maternal mortality: more than 40 % of all global child deaths and more than 50 % of all maternal deaths occur in SSA [2].

Progress in improving health status in SSA has been slow, as measured by progress to the targets of the Millennium Development Goals (MDGs). The MDGs had set targets of reduction, between 1990 and 2015, of 67 % in U5M (MDG number 4) and 75 % in MMR (MDG number 5). With regard to these, the region had achieved, by 2013, only 45 % reduction in U5M and 48 % in MMR [1]. At these rates of progress, SSA as a whole will miss the MDG targets on both of these goals. It has been estimated only nine countries in the region, out of a samples of 36, will achieve their targets on MDG number 4 at the current rates of reduction [3].

Improved health status is not only a goal in its own right but also a prerequisite for the development process since good health is an important form of human capital. Therefore, it is imperative when planning for the post-2015 development agenda to address the question “what can be done to speed up progress in improving health status?” As previous studies show, the amount of resources in the health sector, as measured by public spending on health (PSH), is a potentially important determinant but in itself does not guarantee improved health outcomes. Efficiency in the use of the available resources is necessary to secure the desired improvements [4–6]. Quality of governance (QoG) is considered not only as an important determinant of health outcomes but also of efficiency of public spending on health. This paper intends to examine the role of quality of governance in modifying the effectiveness of PSH in improving health status in Sub Saharan Africa. In doing so it will shed light on the likely extent to which health outcomes in the region can be improved by increasing efficiency in the use of the available resources.

We specifically  focus on SSA for two related reasons. Firstly, it is the region with the worst health outcomes, as highlighted above. Secondly, studies on efficiency of health expenditure have consistently shown that SSA is less efficient in converting health expenditure into improved health status compared to other developing regions. Gupta and Verhoeven employed the Free Disposal Hull technique to estimate the efficiency of government health expenditure on health outcomes in developing countries, controlling for the impact of the level of economic development. They concluded that African economies are inefficient in providing health services relative to their Asian and Western Hemisphere peers [7]. Grigoli and Kapsoli quantified the inefficiency of public health expenditure for emerging and developing economies using a stochastic frontier model that controls for the socioeconomic determinants of health [8]. Their results show that African economies have the lowest efficiency levels. Similar conclusions have been reached by others [9–12].

### Literature review

#### Economic growth and health outcomes

The search for major socioeconomic determinants of good health has been a preoccupation of researchers and policy-makers for decades. Economic growth (sustained increase in income per capita) has consistently been shown to be a major determinant of health outcomes [13, 14]. However, economic growth alone is not adequate to improve health status to desired levels; for example, it is not adequate to reach the MDG targets. As Bokhari [15] argues, the reduction in under-five mortality implied by typical income elasticity of under-five mortality (defined here as the percentage point change in U5M in response to a 1 % change in income) is rather low, and even with good economic growth rates there is a modest effect [15]. This can be illustrated using a pooled income/U5M elasticity of 0.38 for SSA from a systematic review of 24 studies [16], and an economic growth rate of 5 %, the average growth rate for Africa for the past decade [17]. A simple projection is that over the 15 years of MDG implementation from 2000 to 2015, the reduction in U5M would be 25 % against the MDG target of 67 %. That is, economic growth alone is not enough to “produce” good health or reach the MDG.

#### Public spending on health, quality of governance and health outcomes

PSH has long been viewed as a potential complement to economic growth in improving health status. However, there is debate in the literature about the effectiveness of PSH in improving health outcomes. Musgrove summarized most of the earlier cross-country studies on this topic and concluded that while PSH shows little relation to under-five mortality (which is more closely related to nutrition, water and sanitation) there is an association with life expectancy [18]. Several studies have also drawn conclusions of no association between PSH and health outcomes. Applying the instrumental variable (two staged least squares) approach on data from developing countries in the early 1990s, Filmer and Pritchett find that PSH is statistically insignificant and contributes less than 0.2 % when accounting for child mortality differences across countries once they control for income per capita and other covariates [14]. These results are echoed by Wagstaff and Claeson who, when treating PSH as endogenous, found that there is not a statistically significant relationship between PSH and health outcomes [5].

In sharp contrast, other studies find statistically and economically significant results using similar methods to those of Filmer and Pritchett. Using a set of developed and developing countries, Bokhari et al. find the mean elasticity of PSH with respect to U5M and MMR to be −0.33 and −0.50 respectively [15]. Similar results were found by Anyanwu and Erhijakpor who, looking at just SSA countries, found the elasticities of PSH with respect to infant mortality (IMR) and U5M to range from −0.17 to −0.22 and from −0.17 to −0.25, respectively [19]. Gupta et al. also finds PSH to be a good predictor of good health when looking at developing countries and countries in transition. Controlling for the composition of health expenditure, they find the elasticity of PSH with respect to U5M to be −0.29 [20].

Similar conflicts in findings are found in the literature of the influence of PSH on life expectancy. Using panel data from 1995 to 2010 covering 44 countries in SSA, Novignon and colleagues [4] find that health care expenditure significantly influences health status through improving life expectancy at birth. Specifically, they find that a 1 % increase in PSH leads to an improvement in life expectancy by approximately 1 year [4]. Similar findings are obtained by Akinkugbe and Afeikhena [21] for SSA, Middle East and North Africa [21]. There are also contrasting findings. For example, Shaw and colleagues find PSH insignificant in influencing life expectancy for 29 OECD countries for the period 1960 to 1999 [22]. Bayati and colleagues reaches the same conclusion for East Mediterranean region for the period 1995 to 2007 [23].

The differences in the findings of studies reviewed above (and others in the literature) may reflect different sets of countries covered by studies, different time-period considered and different instruments used. More importantly, these differences could also reflect the omission of important confounders, particularly governance-related variables like intra-sectoral allocation, level of efficiency and corruption. Governance not only exerts an independent influence on health outcomes [24–27], but also mediates the impact of PSH on health outcomes. Therefore allocation of funds to the health sector in the setting of poor governance may be insufficient to improve health outcomes [28]. Indeed, poor intra sectoral allocation, poor targeting and inefficiency in delivery are among the reasons for the negligible impact of PSH on health [5, 6]. Novignon and colleagues point out that it is possible for population health to worsen even as health care expenditure increases in the face of resource misallocation and poor management [4]. Therefore, it is very likely that governance may explain the rather surprising lack of association between PSH and outcomes observed in some studies [29]. Rajkumar and Swaroop provide a demonstration of this point. They studied the differences in outcomes of public health spending in the presence of varying levels of quality of governance and report that good governance improves the impact of public spending on child mortality [30]. They found that 1 percentage point increase in the share of PSH in GDP lowers U5M rate by 0.32 % in countries with good governance (as measured by a corruption index), 0.20 % in countries with average governance, and has no impact in countries with weak governance. Lewis has also shown that good governance is important in health care delivery and that returns to investments in health are low where governance issues are not addressed [29].

It has been suggested governance affects health through two main channels. Its indirect effect on national income, (corruption reduces economic growth [31]) and therefore on household income and the determinants of health and directly on the health care sector. In more developed countries governance has a positive effect on health through the healthcare sector while in less developed countries good governance affects health mainly through its indirect impact on income i.e., the income channel predominates [32].

## Methods

### Measures of quality of governance

Governance refers to the manner in which public officials and institutions acquire and exercise the authority to shape public policy and provide public goods and services [33]. The term governance is so multifaceted that several indicators have been developed to try and capture its different dimensions. In this paper, we employ the World Governance Indicators (WGI) by the World Bank, which are defined in Table 1 below.

**Table 1:** Definitions of dimensions of governance

Dimension of Governance	What it captures -
Control of corruption	Perceptions of the extent to which public power is exercised for private gain, including both petty and grand forms of corruption, as well as “capture” of the state by elites and private interests
Government effectiveness	Perceptions of the quality of public services, the quality of the civil service and the degree of its independence from political pressures, the quality of policy formulation and implementation, and the credibility of the government’s commitment to such policies
Political stability	Perceptions of the likelihood that the government will be destabilized or overthrown by unconstitutional or violent means, including politically-motivated violence and terrorism
Regulatory quality	Perceptions of the ability of the government to formulate and implement sound policies and regulations that permit and promote private sector development
Rule of law	Perceptions of the extent to which agents have confidence in and abide by the rules of society, and in particular the quality of contract enforcement, property rights, the police, and the courts, as well as the likelihood of crime and violence
Voice and accountability	Perceptions of the extent to which a country’s citizens are able to participate in selecting their government, as well as freedom of expression, freedom of association, and a free media

Source: World Bank Worldwide Governance Indicators

The WGIs are measured on a scale of scores (in standard deviations) from −2.5 to 2.5. It has been argued that governance scores are inherently subjective and likely biased since they are largely perception-based [12, 30]. However, the construction of WGI measures minimizes this bias since they aggregate findings from a diverse range of surveys conducted by institutes, think tanks, non-governmental organizations and international organizations. Hence, it is reasonable to conclude that the risk of bias due to significant differences between data providers, their aims and their survey/aggregation methods that may be associated with the underlying data is minimized [31]. They are thus a reasonable approximation to the quality of governance. SSA countries generally have poor governance scores on all the dimensions, with mean scores that are lower than the rest of the world regions. The means core for the region, on the different dimensions, is about −0.7.

### Demand for health capital: a simplified Grossman model

Health outcomes can be seen as outcomes of a health production process or function. The health production function used in this paper is based on Grossman’s [34] model of the demand for health. In this model, health is demanded for two purposes: consumption and investment. Health as an investment commodity is an important form of human capital. The core assumption of the model is that an individual inherits an initial stock of health capital that depreciates with time and can be increased through investment. Such gross investment is a function of a household’s own time, market goods like medical care, diet, exercise, recreation and housing, and “environmental variables” like education. An investment in health increases one’s stock of health, which improves health outcomes such as “healthy time”, life expectancy and reduces child mortality [34]. We modify the original Grossman’s micro-level model to a macro-level model and include, among the determinants of health, upstream factors such as public spending on health and quality of governance.

### Estimation strategy

We specified “health production function” in which a country’s health outcomes (H) depend on income per capita (Y), public spending on health (PSH), quality of governance (QoG) and a vector of other socioeconomic status (SES) indicators. That is:

1 $$ H=h\left(Y,PSH,QoG,SES\right) $$

For estimation purposes, we derived from this health production the following multivariate regression of the determinants of health outcomes. For the *i*^*th*^ country in year *t*, we have that

2 $$ {H}_{it}=\alpha +{\beta}_1 \ln {Y}_{it}+{\beta}_2 \ln PS{H}_{it}+{\beta}_3Qo{G}_{it}+\gamma SE{S}_{it}+{\varepsilon}_{it}. $$

H in the present context represents (natural log of) under-five mortality (U5M) and life expectancy at birth (LE). U5M is the probability of dying before the age of five, measured as number of deaths before fifth birthday per 1000 live births. It is regarded as one of the best indicators of child health and the state of primary health care [12, 15]. LE is the number of years an individual can expect to live at birth. Under-five mortality and life expectancy are generally used as aggregate measures of a population’s health status. Income (Y) is measured as gross domestic product per capita (GDPpc), expressed in purchasing power parity terms. GDPpc measures the mean income per person and reflects a country’s state of economic growth/development and is expected to have negative impact on U5M and positive impact on LE. PSH is public spending on health, measured as a percentage of GDP. It is an indicator of public investment in the health sector and in the citizen’s human capital. These expenditures are therefore expected to have negative effects on U5M and positive impact on LE. QoG is the quality of governance and includes five of the six governance indicators taken from the Worldwide Governance Indicators (see Table 1). The excluded indicator is political stability which we thought to be least closely to health outcomes. We estimate eq. 2 with each of these indicators being used, in turn, as a measure of quality of governance. SES is a vector of socio-economic status. It includes female literacy, sanitation, immunization, urbanization and number of physicians. QoG and SES are expected to have negative effects onU5M and positive impact on LE.

Income and public spending on health are in natural log. This serves several purposes: it takes care of the nonlinearity in the relationship between these variables and health outcomes; provides nice interpretation of the coefficients as elasticities in the case of U5M (which is also in natural log form) and semi-elasticity in the case of LE (which is in levels); and, makes the results easily comparable to those of previous studies.

To account for the modifying effect of quality of governance on the impact of PSH on health outcomes, we introduced an interaction term between them. Therefore, we also estimated the following regression equation:

3 $$ {H}_{it}=\alpha +{\beta}_1 \ln {Y}_{it}+{\beta}_2 \ln PS{H}_{it}+{\beta}_3Qo{G}_{it}+{\beta}_4\left(Qo{G}_{it}* \ln PS{H}_{it}\right)+\gamma SE{S}_{it}+{\varepsilon}_{it} $$

In order to further elaborate on the modifying effect of quality of governance, we examine the total effect of PSH on health outcomes, both directly and indirect through QoG. To do this, it must be observed that the overall elasticity of public health spending with respect to health outcomes from regression eq. (3) is:

4 $$ \frac{\partial {H}_{it}}{\partial \ln PS{H}_{it}}={\beta}_2+{\beta}_4*Qo{G}_{it} $$

That is, the overall effect of PSH on health outcomes is the sum of the direct effect (*β*_2_) and indirect effect through quality of governance (*β*_4_ * *QoG*_*it*_). It is evident from eq. (4) that the impact of health spending on health outcomes is dependent on the quality of governance. We calculate this effect at three levels: the mean score of governance for each indicator (mean score), at one standard deviation below (lower score) and above the mean (upper score).

Several statistical issues may arise in estimating regression eqs. (2) and (3). Firstly, given the panel dataset used, we have to verify presence of country-specific fixed effects. We employ the Hausman test to choose between random effects and fixed effects estimation for each regression that was run. Secondly, heteroskedasticity and autocorrelation can lead to insignificant estimates. To take care of heteroskedasticity and autocorrelation, we use clustering which renders the standard errors robust to these two problems. Thirdly, the relationship between PSH and health outcomes and between income and health outcomes can run in both directions. That is, changes in PSH or income may lead to, and/or react to, changes in health outcomes. That is, health spending and income are potentially endogenous and this may bias the estimates. We therefore used the instrumental variable (IV) approach (i.e., two staged least squares) to control for this endogeneity (reverse causality). Following Filmer and Pritchett, we instrument PSH by military spending in neighbouring countries [14]. Following Bokhari and colleagues we use consumption-investment ratio as the instrument for GDP per capita [15]. We test validity of these instruments Sargan’s over-identification test, which tests whether the instrument are uncorrelated with the error term.

We use data from 43 Sub Saharan African countries from 1995 to 2011, extracted from the World Bank’s World Development Indicators and Worldwide Governance Indicators databases. This choice of sample period was necessitated by the availability of quality of governance data.

## Results

Based on the Hausman specification tests, the fixed effects model was favoured over the random effects model. The results presented in this section are thus based on the fixed effects model. Table 2 below presents results of eq. (2) with under-five mortality as the dependent variable. The top panel of Table 2 shows the estimates for eq. (2) with each of the five indicators of governance used in turn. Guided by previous studies, the vector of socio-economic status included female literacy, sanitation, access to safe water, immunisation, urbanisation rates and physicians per capita. Of these, only female literacy and sanitation were significant at times. Given the statistical dictates of a parsimonious model in explaining any phenomenon, and the mixed results from previous studies regarding the significance of the socioeconomic variables, there was no obvious gain for including them. Therefore, with the exception of female literacy and sanitation, we did not include them.

**Table 2:** Regression results for Under-five mortality

OLS										
GDP per capita	−0.381^**^	(0.136)	−0.391^**^	(0.138)	−0.379^**^	(0.134)	−0.371^**^	(0.132)	−0.388^**^	(0.136)
Public Health Expenditure	−0.103	(0.058)	−0.104	(0.057)	−0.102	(0.058)	−0.095	(0.056)	−0.110	(0.059)
Female literacy	−0.008^***^	(0.001)	−0.008^***^	(0.001)	−0.008^***^	(0.001)	−0.008^***^	(0.001)	−0.008^***^	(0.001)
Sanitation	−0.297	(0.174)	−0.301	(0.173)	−0.297	(0.177)	−0.295	(0.179)	−0.292	(0.181)
Government Effectiveness	−0.002	(0.070)								
Regulatory Quality			0.042	(0.074)						
Control of Corruption					−0.006	(0.065)				
Rule of Law							−0.063	(0.089)		
Voice and Accountability									0.043	(0.063)
Constant	8.894^***^	(0.881)	9.002^***^	(0.889)	8.876^***^	(0.851)	8.791^***^	(0.850)	8.948^***^	(0.866)
Adjusted R-squared	0.67		0.68		0.67		0.68		0.68	
F	36.31		37.33		36.53		38.39		38.34	
Observations	374		374		374		374		374	
IV										
GDP per capita	−0.487^***^	(0.082)	−0.484^***^	(0.081)	−0.479^***^	(0.083)	−0.450^***^	(0.080)	−0.489^***^	(0.082)
Public Health Expenditure	−0.094^**^	(0.030)	−0.101^***^	(0.030)	−0.098^**^	(0.031)	−0.087^**^	(0.029)	−0.114^***^	(0.031)
Female literacy	−0.009^***^	(0.001)	−0.009^***^	(0.001)	−0.009^***^	(0.001)	−0.009^***^	(0.001)	−0.009^***^	(0.001)
Sanitation	−0.046	(0.068)	−0.078	(0.064)	−0.093	(0.065)	−0.062	(0.063)	−0.072	(0.065)
Government Effectiveness	−0.096^*^	(0.047)								
Regulatory Quality			−0.108^*^	(0.043)						
Control of Corruption					−0.015	(0.037)				
Rule of Law							−0.183^***^	(0.046)		
Voice and Accountability									0.082^*^	(0.040)
Sargan (*p*-value)	0.369		0.342		0.241		0.313		0.205	
Adjusted R-squared	0.68		0.68		0.67		0.70		0.68	
F	100.37		102.02		97.60		108.58		100.46	
Observations	223		223		223		223		223	

Standard errors in parentheses

^*^ *p* < 0.05, ^**^ *p* < 0.01, ^***^ *p* < 0.001

The results in the top panel are in conformity with those in the literature regarding the negative impact of income and female literacy on child mortality. The income elasticity of under-five mortality ranges from −0.37 to −0.39, magnitudes which are in tandem with previous studies such as those by Filmer and Pritchett [14], Gupta et al. [20] and summarized by O’Hare and colleagues [16]. These elasticities mean that if income increases by, say, 10 % then there will be a decrease in under-five mortality of between 3.7 % and 3.9 %, holding all else constant. Female literacy is also significant statistically but the elasticities are smaller relative to those of income. Public spending on health is insignificant. Similarly, sanitation and measures of quality of governance are insignificant.

These results may however be misleading in the presence of endogeneity of income and public health spending. To correct the endogeneity bias, we use the instrumental variable estimation technique. The results are shown in the bottom panel of Table 2. Income and female literacy are again negative and statistically significant. It is noteworthy, however, that while the elasticity of female literacy has almost stayed the same, the income elasticity of under-five mortality has increased to the range −0.45 to −0.49. That is, if income increases by, say, 10 % then under-five mortality will fall by between 4.5 % and 4.9 %, holding all else constant. This means that, in the presence of endogeneity, the estimates for income elasticities were biased downwards.

Further, with endogeneity bias corrected, public spending on health becomes a significant determinant of child mortality. Its elasticity ranges from −0.09 to −0.11, depending on the measure used for QoG. That is, if PSH increases by, say, 10 % then under-five mortality will fall by between 0.9 % and 1.1 %, holding all other factors constant. From these results, income elasticity of U5M is thus roughly four times as high as PSH elasticity of U5M. This finding supports the widely held argument that income is the more dominant determinant of the two. Possible reasons for the lower effect of public spending includes poor targeting and/or institutional inefficiencies such as leakage in public spending and weak institutional capacity and substitution effect between public and private (out of pocket) spending on health [30].

With the two-staged least squares, all the indicators of governance except control of corruption are significant, implying that quality of governance is an important influence on  child mortality. The estimated coefficients range from −0.08 to −0.18, meaning that a unit (one standard deviation) improvement in governance leads to a decline in child mortality of between 8 % and 18 %. Improving quality of governance, therefore, could have  a considerable direct impact in reducing child mortality. These results are consistent with those of previous studies by Lin et al. [24], Kaufmann et al. [26] and Helleröd et al. [27].

As pointed out in the methods section, the instrumental variable approach provides a good remedy for endogeneity. However, this approach works only when the instruments are valid. Otherwise, the remedy may be worse than the problem. The instruments used are valid since the Sargan’s statistic is insignificant as per the *p*-values shown in the third-from-last row of the table. The instrumental variable results can therefore be taken with confidence.

The analysis above is repeated for life expectancy. The results are shown in Table 3 below. For the instrumental variable regression, income has coefficients of between 2.01 and 2.83. This means that an increase in income, of say 10 %, adds between 0.2 years (over two months) and 0.3 years (nearly four months) to life expectancy. Increases in PSH also adds to life expectancy, albeit a lower  contribution relative to that of income. Its coefficients are in the range of 0.35 to 0.60. The impact of governance is very varied, ranging from 1.29 to 4.45. That is, a unit improvement in governance adds between 1 and 4 years to life expectancy, all else being equal.

**Table 3:** Regression results for life expectancy

OLS										
GDP per capita	3.582^*^	(2.068)	3.430	(2.215)	3.103^*^	(1.548)	3.254^*^	(1.688)	3.510^*^	(2.080)
Public Health Expenditure	0.195	(0.714)	0.337	(0.713)	0.261	(0.706)	0.092	(0.687)	0.232	(0.784)
Female literacy	0.101^***^	(0.030)	0.093^***^	(0.029)	0.097^***^	(0.029)	0.101^***^	(0.030)	0.093^***^	(0.029)
Sanitation	2.909^*^	(1.635)	3.494^**^	(1.574)	3.525^**^	(1.519)	3.474^**^	(1.520)	3.663^**^	(1.450)
Government Effectiveness	2.007^**^	(0.899)								
Regulatory Quality			0.833	(1.283)						
Control of Corruption					1.190	(0.948)				
Rule of Law							2.362^*^	(1.234)		
Voice and Accountability									0.776	(1.151)
Constant	14.178	(14.12)	12.993	(15.23)	15.204	(12.85)	14.896	(13.32)	11.851	(13.77)
Adjusted R-squared	0.45		0.44		0.44		0.46		0.44	
F	25.01		28.15		25.76		24.45		24.85	
Observations	372		372		372		372		372	
IV										
GDP per capita	2.017^*^	(1.267)	1.876	(1.616)	2.521^*^	(1.683)	2.052^*^	(1.602)	2.825^**^	(1.688)
Public Health Expenditure	0.601^***^	(0.264)	0.803	(0.595)	0.622^***^	(0.201)	0.349^*^	(0.590)	0.690	(0.637)
Female literacy	0.131^***^	(0.020)	0.112^***^	(0.020)	0.125^***^	(0.021)	0.134^***^	(0.020)	0.122^***^	(0.021)
Sanitation	0.551	(1.364)	1.832	(1.281)	2.306^*^	(1.321)	1.550	(1.268)	2.342^*^	(1.348)
Government Effectiveness	3.602^***^	(0.942)								
Regulatory Quality			3.522^***^	(0.847)						
Control of Corruption					1.289^*^	(0.743)				
Rule of Law							4.448^***^	(0.923)		
Voice and Accountability									0.124	(0.817)
Sargan (*p*-value)	0.481		0.237		0.174		0.290		0.187	
Adjusted R-squared	0.33		0.34		0.29		0.36		0.28	
F	28.70		29.55		24.99		31.44		24.04	
Observations	223		223		223		223		223	

Standard errors in parentheses

^*^ *p* < 0.10, ^**^ *p* < 0.05, ^***^ *p* < 0.01

To take into account the modifying effect of quality of governance on PSH, we run the regression with the interaction between the two factors (eq. 3). To economise on space, we only report regression results of the instrumental variable approach. Table 4 show results from regression (eg. 3) for under-five mortality. The results are broadly similar to those of regressions without the interaction term. Our focus in these regressions is on the interaction terms. For under-five mortality, significant interactions exist between public spending on health and government effectiveness, rule of law and, voice and accountability.

**Table 4:** Instrumental Variable Regression results for Under-five mortality - with interactions

GDP per capita	−0.449^***^	(0.085)	−0.461^***^	(0.083)	−0.474^***^	(0.090)	−0.427^***^	(0.082)	−0.526^***^	(0.082)
Public Health Expenditure	−0.128^***^	(0.037)	−0.126^***^	(0.036)	−0.100^**^	(0.033)	−0.110^**^	(0.035)	−0.071^*^	(0.036)
Female literacy	−0.009^***^	(0.001)	−0.009^***^	(0.001)	−0.009^***^	(0.001)	−0.009^***^	(0.001)	−0.009^***^	(0.001)
Sanitation	−0.071	(0.070)	−0.094	(0.066)	−0.095	(0.067)	−0.072	(0.063)	−0.051	(0.065)
Government Effectiveness	−0.063^*^	(0.034)								
Public Health Expenditure * Government effectiveness	−0.069^*^	(0.041)								
Regulatory Quality			−0.062	(0.057)						
Public Health Expenditure * Regulatory Quality			−0.056	(0.047)						
Control of Corruption					−0.009	(0.057)				
Public Health Expenditure * Control of Corruption					−0.007	(0.044)				
Rule of Law							−0.146^**^	(0.056)		
Public Health Expenditure * Rule of Law							−0.054^*^	(0.032)		
Voice and Accountability									−0.047^*^	(0.023)
Public Health Expenditure * Voice and Accountability									−0.087^*^	(0.038)
Sargan (*p*-value)	0.371		0.379		0.242		0.348		0.244	
Adjusted R-squared	0.68		0.68		0.67		0.70		0.68	
F	84.55		85.41		80.91		90.86		86.31	
Observations	223		223		223		223		223	

Standard errors in parentheses

^*^ *p* < 0.05, ^**^ *p* < 0.01, ^***^ *p* < 0.001

For these interaction terms, a one unit improvement in the indicator of governance, leads to between 6 to 8 % increase in the impact of public health spending in reducing child mortality. This suggests that improving governance enhances the influence of PSH on child mortality. That is, if governance improved the same amount of resources spent on the health sector could reduce U5M further. This is shown in Table 5 where the effect on under-five mortality of the same public expenditure is assessed at various levels of quality of governance, using eq. (4). We call these levels lower (one standard deviation below the mean score), mean (mean score of a measure of governance) and upper (one standard deviation above the mean score). For countries with good governance (i.e., above the mean score), the elasticity of under-five mortality with respect to public health spending is about −0.17 to −0.19, while in countries with poor governance the elasticity is about −0.09. This result suggests that improved quality of governance improve the efficiency with which public spending on health can be converted to better health outcomes. Our results concur with those of Rajkumar and Swaroop [30]. For a set of 91 developed and developing countries, they find that a 1 percentage point increase in the share of public health spending in GDP lowers the under-5 mortality rate by 0.32 % in countries with good governance (as measured by a corruption index and bureaucratic quality), 0.20 % in countries with average governance, and has no impact in countries with weak governance. Our results are slightly lower, probably because we have used one developing region where governance scores are particularly low.

**Table 5:** effect of PSH on U5M at different levels of QoG

	lower^a^	mean^b^	upper^c^
Government effectiveness	−0.09346	−0.13555	−0.17764
rule of law	−0.09256	−0.13741	−0.18226
voice and accountability	−0.09298	−0.14321	−0.19344

Notes: ^a^elasticity computed at one standard deviation below the sample mean score; ^b^elasticity computed at the sample mean score; ^c^elasticity computed at one standard deviation above the sample mean score

Turning to life expectancy, significant interactions exist between public spending on health and control of corruption, rule of law and, voice and accountability as shown in Table 6 below. For these interaction terms, a one unit improvement in the indicator of governance increases the impact of public health spending in lengthening life expectancy by 0.02 years and 0.031 years. That is, the same amount of resources spent on the health sector could increase life expectancy if quality of governance improves. This is shown in Table 7 where the effect on life expectancy of the same public expenditure is assessed at various levels of quality of governance, using eq. (4). For countries with good governance (i.e., above the mean score), the semi-elasticity of life expectancy with respect to public health spending is about 6, while in countries with bad governance the elasticity is about 3.

**Table 6:** Instrumental Variable Regression results for Life expectancy- with interactions

GDP per capita	2.825^*^	(1.695)	2.478	(1.649)	3.048^*^	(1.801)	1.931	(1.632)	3.120^*^	(1.654)
Public Health Expenditure	0.968	(0.744)	0.645	(0.718)	0.718^*^	(0.457)	1.187^*^	(0.696)	1.593^**^	(0.721)
Adult literacy	0.128^***^	(0.020)	0.108^***^	(0.020)	0.116^***^	(0.021)	0.131^***^	(0.020)	0.113^***^	(0.020)
Sanitation	0.020	(1.396)	1.398	(1.301)	1.605	(1.343)	1.161	(1.265)	1.617	(1.308)
Government Effectiveness	4.906^***^	(1.247)								
Public Health Expenditure * Government effectiveness	−1.427	(0.901)								
Regulatory Quality			4.749^***^	(1.139)						
Public Health Expenditure * Regulatory Quality			1.504	(0.941)						
Control of Corruption					3.219^***^	(1.142)				
Public Health Expenditure * Control of Corruption					1.946^**^	(0.882)				
Rule of Law							5.827^***^	(1.106)		
Public Health Expenditure * Rule of Law							2.009^**^	(0.914)		
Voice and Accountability									2.214^**^	(0.943)
Public Health Expenditure * Voice and Accountability									3.080^***^	(0.769)
Sargan (*p*-value)	0.441		0.179		0.122		0.215		0.198	
Adjusted R-squared	0.33		0.34		0.30		0.37		0.33	
F	24.50		25.23		22.02		27.49		24.16	
Observations	223		223		223		223		223	

Standard errors in parentheses

^*^ *p* < 0.05, ^**^ *p* < 0.01, ^***^ *p* < 0.001

**Table 7:** Effect of PSH on LE at different levels of QoG

	Lower^a^	Mean^b^	Upper^c^
Control of corruption	3.2901	4.45815	5.6262
rule of law	2.9742	4.2417	5.5092
voice and accountability	2.9859	4.4055	5.8251

Notes: ^a^semi-elasticity computed at one standard deviation below the sample mean score; ^b^semi- elasticity computed at the sample mean score; ^c^semi-elasticity computed at one standard deviation above the sample mean score

## Discussion

On the whole, the results show that public spending on health is an important determinant of health outcomes in SSA. The direct elasticity of PSH with respect to under-five mortality is between −0.09 and −0.11 while its semi-elasticity with respect to life expectancy is between 0.35 and 0.60. These results broadly confirm the findings of those studies by Gupta [20], Bokhari [15], Anyanwu [19] and Novignon [4] who found PSH to be significant  in influencing health outcomes. It is also noteworthy that the effect on health outcomes implied by these figures is small relative to the effect of income. For example, our results suggest that income elasticity of U5M is roughly four times as high as PSH elasticity of U5M. Possible reasons for the lower effect of public spending includes poor targeting and/or institutional inefficiencies such as leakage in public spending and weak institutional capacity and substitution effect between public and private (out of pocket) spending on health [30].

In addition, the impact of PSH on health outcomes is significantly mediated by QoG. Specifically, government effectiveness, rule of law and accountability mediates the impact of PSH on U5M while control of corruption, rule of law and accountability mediates impact of PSH on LE. The results are summarised in Table 8 below.

**Table 8:** Effect of PSH on U5M and LE at different levels of QoG – key findings

	Under-five mortality		Life expectancy	
	Lower^a^	Upper^b^	Lower^c^	Upper^d^
Government effectiveness	−0.09346	−0.17764		
Rule of law	−0.09256	−0.18226	2.9742	5.5092
Voice and accountability	−0.09298	−0.19344	2.9859	5.8251
Control of corruption			3.2901	5.6262

The overall effect of the same amount of PSH on health outcomes is dependent on the quality of governance in a country; it will have more impact in a country with good governance and minimal impact in a poorly governed country in SSA. The same increase in PSH is twice as effective in reducing U5M and increasing LE in countries with higher QoG as with lower QoG, as shown in the table above

Notes: ^a^elasticity computed at one standard deviation below the sample mean score; ^b^semi-elasticity computed at one standard deviation above the sample mean score; ^c^semi-elasticity computed at one standard deviation below the sample mean score; ^d^semi-elasticity computed at one standard deviation above the sample mean score

For countries with higher quality of governance (i.e., above the mean score), the overall elasticity of under-five mortality with respect to public health spending is about -.17 to -.19, while in countries with lower quality of governance the elasticity is about −0.09. For life expectancy, countries with higher quality of governance (i.e., above the mean score), the overall semi-elasticity of life expectancy with respect to public health spending is about 6, while in countries with bad governance the elasticity is about 3. These results suggest that the same amount of resources is twice as effective in improving health outcome in countries with higher quality of governance as in those countries with lower quality of governance.

These findings provide one possible explanation for the surprising result that public spending on health often does not yield the expected improvement in health outcomes. As highlighted in the literature review, some studies, including the influential paper by Filmer and Pritchett [14], have found insignificant or an extremely small impact of public spending. Our findings show that the quality of governance may be the explanation and this is supported by Rajkumar and Swaroop [30], who studied the impact of public spending on development outcomes, including under-five mortality, at different levels of quality of governance. Their findings show that while public spending on health is effective in reducing U5M in well-governed countries, it has virtually no impact in poorly governed countries. Probable explanations for this observation is that improving QoG leads to better allocation of resources, better targeting and enhances more efficient use of available resources. As Wagstaff and Claeson [5] point out, better governance leads to better policies and more efficient institutions, hence strengthening the link between PSH and health outcomes. Similarly, Lewis [29] concludes from a study of 119 developing countries that governance is important in ensuring effective health care delivery and that returns to investments in health are low where governance issues are not addressed.

Apart from mediating the effectiveness of PSH, QoG has its own direct effect on health outcomes. All the indicators of governance except control of corruption are directly significant in reducing under-five mortality, with elasticities ranging from −0.09 to −0.11 while for life expectancy, all indicators except government effectiveness are significant, with semi elasticities ranging from 1.29 to 4.45. The importance of quality of governance for health is echoed in the literature by Kaufmann et al. [26], Wagstaff and Claeson [5], Lewis [29], Holmberg and Rothsten [35] and Halleröd et al. [27] among others. Population health can be related to QoG in several ways. Firstly, since a country’s QoG is positively related to economic performance, high QoG should result in more economic growth, which should imply better food, better housing, access to safe water and sanitation, less strenuous working conditions, fewer people living under destitute conditions and so forth [35]. Secondly, quality health care service delivery is dependent on quality of policies and administrative institutions, both of which improve with QoG. Higher QoG should result in more efficient use and better-targeting of resources in healthcare delivery and thus better population health.

## Conclusions

Public health spending improves health outcomes. Its impact is mediated by quality of governance, having the higher impact on health outcomes in countries with better governance and lower impact in countries with poor governance. This provides one possible explanation for the insignificant or extremely small impact of public spending observed in some studies, namely; that inefficiency of public spending may be, in actual fact, a result of poor governance. Governance is important in ensuring effective health care delivery and returns to investments in health are low where governance issues are not addressed. It is therefore imperative that SSA governments improve the quality of governance as one way of improving health outcomes.

### Ethics approval

We used public and freely available data, ethical approval was not required.

## Abbreviations

GDP: Gross Domestic Product
QoG: Quality of Governance
LE: Life Expectancy
MMR: Maternal Mortality Ratio per 100,000 deliveries
PSH: Public Spending on Health
U5M: Under five mortality rate per 1000 livebirths
SSA: Sub Saharan Africa
